# Transesterification of Lactic Acid Oligomers with Ethanol, a Way to Anhydrous Ethyl Lactate: A Kinetic Study

**DOI:** 10.3390/molecules23082044

**Published:** 2018-08-15

**Authors:** Silvestr Figalla, Jaroslav Petrůj, Tereza Švestková

**Affiliations:** 1 Materials Research Centre, Brno University of Technology, 612 00 Brno, Czech Republic; 2 Faculty of chemistry, Institute of Material Chemistry, Brno University of Technology, 612 00 Brno, Czech Republic; petruj@fch.vutbr.cz; 3 Faculty of chemistry, Institute of Environmental Chemistry, Brno University of Technology, 612 00 Brno, Czech Republic; xcsvestkovat@fch.vutbr.cz

**Keywords:** ethyl lactate, transesterification, kinetics, lactic acid, oligomerization

## Abstract

A new method for the preparation of anhydrous ethyl ester of lactic acid was studied. The selected method is based on catalytic transesterification of lactic acid oligomers, which were prepared for this purpose by autocatalytic oligomerization of lactic acid. In this work, a kinetic model for the case of catalytic alcoholysis of oligoesters was derived assuming a first-order reaction and equimolar content of reactants in the reaction mixture. The model makes it possible to obtain the values of the reaction rate and equilibrium constants and the equilibrium alcohol concentration by regression analysis at one time. The model was verified by measuring the rate of consumption of ethanol over the time at various reaction temperatures with anhydrous FeCl_3_ as the catalyst. The reaction was studied at overpressure under autogenous conditions in the temperature range of 100–180 °C. For the catalyst concentration of 1 mol %, the activation energy value was 64.35 kJ·mol^−1^. The dependence of equilibrium composition and rate constant on the temperature was obtained. The derived model is generally applicable to all first-order equilibrium reactions. The presumption is that the forward and reverse reactions are of the same order and have the same stoichiometry and equivalent amounts of reactants at the beginning of the reaction.

## 1. Introduction

Esters of lactic acid are an important group of derivates with increasing use as bio-solvents. The reason for this is that the use of these esters has a small environmental impact owing to their partial or complete renewability of feedstock, miscibility with water and organic phase, and their great dissolving power [1]. Ethyl lactate finds use as a solvent in organic synthesis, enzymatic reactions, and as an extraction agent in pharmacology [2,3,4]. Large-volume applications can be found in the consumer and industrial sectors. It may be used as a green solvent in painting systems, printing inks, and graffiti removers [1,5]. Ethyl lactate is a powerful nonvolatile degreasing agent [6]. It can be used as an ecological extraction agent for soil decontamination and in the electrotechnical industry to remove fluxes after soldering of printed circuit boards [7,8]. A potential new use may be found in polymer chemistry for low and high molecular weight lactic acid polymers via transesterification synthesis [9]. The most promising lactic acid ester in terms of no toxicity and a fully renewable raw material base is ethyl lactate. Its wider application as a bio-solvent is still hindered due to difficult production and the resulting higher price compared to classic solvents [10]. The preparation of ethyl lactate is complicated by the auto-oligomerization of lactic acid and the esters themselves. Ethyl lactate forms azeotrope with water when the water content is 29.7% [11]. Water is brought into the reaction medium with the solution of crude lactic acid (usually 80–90% aqueous solution) and as a by-product of esterification. The inconvenient order of volatilities of components in the reaction mixture complicates the recovery of pure anhydrous ester by distillation. The cascade of three distillation columns is required to completely separate the reaction mixture. These drawbacks partly eliminate the preparation method based on reactive distillation or pervaporation membrane hybrid systems. However, the design of these devices, the possibilities of their control, and the design solution are still uncommon [12,13,14]. As an alternative, based on conventional unit operations, we suggest the preparation of lactic acid esters by the transesterification of oligomeric lactic acid with anhydrous alcohols, while water from the reaction system has already been removed during prior oligomerization of aqueous lactic acid solution. The autocatalytic oligomerization can lead to a sufficient degree of condensation. The product of transesterification of oligomers by alcohol is the anhydrous mixture of esters and their oligomers from which ester can be easily obtained by distillation. Removing water from the system prior to the esterification has a positive effect on the chemical balance with respect to the product. The ester is formed with good yield even with an equimolar content of the reactants. Previously published works on the preparation of lactic acid esters show the behavior of the esterification mixture with a significant alcohol excess in order to displace the esterification equilibrium in the product direction [15,16,17,18]. With equimolar or only a small excess of alcohol in the esterification mixture, the kinetics of transesterification and the equilibrium composition are markedly affected by the chemical equilibrium. Therefore, this publication focuses on the derivation of a necessary kinetic model and subsequent experimental verification of the theoretical assumptions. The model allows the transesterification rate and equilibrium constants with respect to ethanol to be obtained. Experimental verification is demonstrated by the preparation of anhydrous ethyl lactate by the transesterification of oligomeric lactic acid at an equivalent content of reactants. Anhydrous FeCl_3_ was used as a catalyst, as previously reported for the alcoholysis of high molecular weight poly(lactic acid) [19]. This ecological catalyst belongs to the broad group of Lewis acids that are commonly used in organic synthesis [20,21]. The mechanism of catalytic effect of Lewis acids in esterification and transesterification reactions has been described previously [22]. Other potentially useful common substances in this group are, for example, chlorides, acetates, and perchlorates of Sn^2+^, Al^3+^, Fe^3+^, Zn^2+^, and Sb^3+^ [23,24,25,26]. Pre-oligomerization limits the amount of water in the esterification environment prior to the addition of the catalyst. This allows the use of these catalysts, which undergo rapid hydrolysis and a change of catalytic effect, in an aqueous medium. Lewis acids can replace the commonly used strong organic and inorganic acids, thereby reducing the environmental impact of a given esterification process [27].

## 2. Results and Discussion

### 2.1. Derivation of the Kinetic Model

**Figure 1:**
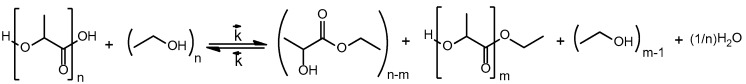
Full reaction scheme for the transesterification of oligomeric LA to ethyl lactate including equilibrium (free) ethanol, water liberated by end-group esterification, and the oligomeric ester fraction (valid for an equimolar mixture of ethanol and lactate units in the oligomer and n > m).

Obtained concentration vs. time data points during the alcoholysis at different reaction temperatures serve to verify the correctness of the proposed kinetic model derived for the case of alcoholysis in a homogeneous reaction medium with homogeneous catalysis. The model is derived for an equimolar ratio of reactants. In this case, the course of the reaction already significantly influences the equilibrium character of alcoholysis (transesterification). The model was designed to find a simple method for the determination of rate constant, equilibrium constant, and equilibrium composition. As previously proven, the catalytic alcoholysis of polyesters follows first-order kinetics [28]. The model assumes the same reaction order for the reverse reaction. Because of a large number of alcoholysis oligomer products (the ester and its oligomers), the kinetic model was derived for the rate of ethanol consumption. From the viewpoint of ethanol consumption, the process is unambiguous and can be understood in more general terms as the reaction of alcohol with ester bonds. The influence of oligomer end groups on the reaction rate and equilibrium composition (water released by esterification of terminal carboxyl) was neglected. The reaction scheme of lactic acid oligomer alcoholysis is shown in Figure 1.

### 2.2. List of Symbols

$x_{0}=$ initial alcohol concentration, $x_{e}=$ alcohol concentration at equilibrium, x= alcohol concentration at time *t*, $\vec{k}=$ rate constant of forward reaction (alcohol consumption), $\overset{\leftarrow }{k}=$ rate constant of reverse reaction (alcohol regeneration), $\vec{r}=$ rate of forward reaction (alcohol consumption), $\overset{\leftarrow }{r}=$ rate of reverse reaction (alcohol regeneration),$\overset{↔}{k}=$ overall rate constant, $\overset{↔}{r}=$ overall reaction rate, K= equilibrium constant, T= thermodynamic temperature, R= universal gas constant

The overall reaction rate is:

(1) $$\overset{↔}{r}=-\vec{k}\cdot \left(x_{0}-x\right)-\overset{\leftarrow }{k}\cdot x$$

At equilibrium, it is:

(2) $$t=\infty ;\;\vec{r}=\overset{\leftarrow }{r}=\overset{↔}{r};\;x=x_{e}$$

The substitution in Equation (1) with (Equation (2)) for the equilibrium state:

(3) $$-\vec{k}\cdot \left(x_{0}-x_{e}\right)=\overset{\leftarrow }{k}\cdot x_{e}$$

The expression of the rate constant of the reverse reaction $\overset{\leftarrow }{k}$ of Equation (3) and the substitution in Equation (1) for the total rate:

(4) $$\overset{↔}{r}=-\vec{k}\cdot \left(x_{0}-x\right)+\frac{\vec{k}\cdot \left(x_{0}-x_{e}\right)}{x_{e}}\cdot x$$

By the rearrangement of Equation (4):

(5) $$\overset{↔}{r}=\frac{\vec{k}\cdot x_{0}}{x_{e}}\cdot \left(x-x_{e}\right)$$

The differentiation of Equation (5):

(6) $$\overset{↔}{r}=-\frac{dx}{dt}=\frac{\vec{k}\cdot x_{0}}{x_{e}}\cdot \left(x-x_{e}\right)$$

The separation of variables in Equation (6):

(7) $$-\frac{\vec{k}\cdot x_{0}}{x_{e}}\cdot dt=\frac{1}{\left(x-x_{e}\right)}dx$$

The integration of Equation (7) for $t=0\;\to \;x=x_{0}\;;\;t=\infty \;\to \;x=x_{e}$

(8) $$-\frac{\vec{k}\cdot x_{0}}{x_{e}}\int dt=\int \frac{1}{\left(x-x_{e}\right)}dx$$

Equation (8) for boundary conditions the following equation:

(9) $$-\frac{\vec{k}\cdot x_{0}}{x_{e}}\cdot t=\ln\left(\frac{x-x_{e}}{x_{e}}\right)-\ln\left(\frac{x_{0}-x_{e}}{x_{e}}\right)$$

The equilibrium constant equals:

(10) $$K=\vec{\frac{k}{\overset{\leftarrow }{k}}}=\frac{\left[product\right]}{\left[reactant\right]}=\frac{x_{0}-x_{e}}{x_{e}}$$

Substituting Equation (10) into Equation (9):

(11) $$-\frac{\vec{k}\cdot x_{0}}{x_{e}}\cdot t=\ln\left(\frac{x-x_{e}}{x_{e}}\right)-lnK$$

By the rearrangement of Equation (11) into the common linear equation y=a·t+b:

(12) $$\ln\left(\frac{x-x_{e}}{x_{e}}\right)=-\frac{\vec{k}\cdot x_{0}}{x_{e}}\cdot t+\ln K$$

Only the value of the total velocity constant of the equilibrium reaction is experimentally accessible. The value is the sum of the forward and reverse rate constants where the former represents the member of Equation (12):

(13) $$\left(\vec{k}+\overset{\leftarrow }{k}\right)=\frac{\vec{k}\cdot x_{0}}{x_{e}}=\overset{↔}{k}$$

Substituting Equation (13), one obtains the equation for the total rate of the first-order equilibrium reaction. By expressing this equation in a linearized form, we obtain a linear equation where the slope is the total speed constant $\overset{↔}{k}$ and the member (ln*K*) is the intercept of this equation:

(14) $$\ln\left(\frac{x-x_{e}}{x_{e}}\right)=-\overset{↔}{k}\cdot t+\ln K$$

By the exponentiation and rearrangement, we get the final relation for the concentration of reactant ***x*** at time *t*, including the equilibrium reaction constant *K*, the total velocity constant $\overset{↔}{k}$, and the content of reactant in the equilibrium ***x_e_***:

(15) $$x=x_{e}\cdot \left[1+K\cdot e^{-\overset{↔}{k}\cdot t}\right]$$

### 2.3. Lactic Acid Oligomerization

**Figure 2:**
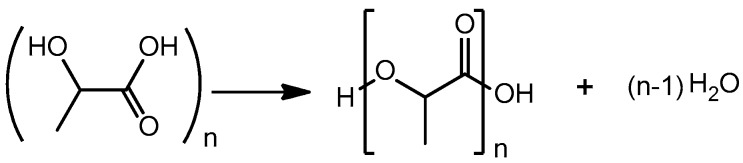
Scheme of autocatalytic lactic acid oligomerization.

**Figure 3:**
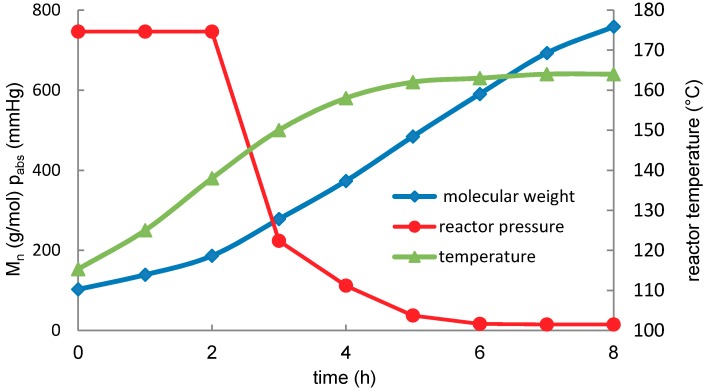
Pressure, temperature, and average molecular weight change in time during the autocatalytic oligomerization of lactic acid.

The autocatalytic oligomerization of lactic acid was carried out according to Figure 2 under the conditions shown in Figure 3. The autocatalytic condensation of lactic acid allows virtually linear growth of the molecular weight to a degree of condensation of $\bar{\mathrm{PD}}\approx 11$, ($\bar{M_{n}}=758$ g·mol^−1^). The condensation rate is initially limited by the rate of condensate (water) removal and is thus mainly dependent on the temperature and pressure during the condensation. The conclusions on the oligomerization process cannot be generalized because they are mainly dependent on the experimental conditions.

The amount of water removed during the oligomerization is equal to the sum of the water contained in the crude lactic acid–water solution and the water produced by the condensation of lactate units. The total relative reduction of the water content (*RW*) in the reaction medium as compared to direct esterification of lactic acid water solution with ethanol is expressed in Equation (16).

(16) $$RW=\left[1-\frac{M_{H_{2}O}}{\bar{PD}\cdot \left(M_{H_{2}O}-M_{\mathrm{LA}}+\frac{M_{\mathrm{LA}}}{x_{\mathrm{LA}}}\right)}\right]\times 100\;\left[\%\right]$$

where $M_{H_{2}O}=$ water molecular weight, $M_{\mathrm{LA}}=$ lactic acid molecular weight, $x_{\mathrm{LA}}=$ lactic acid mass fraction in its water solution.

**Figure 4:**
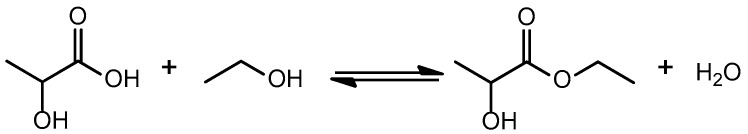
Idealized reaction scheme for direct esterification of lactic acid with ethanol (formation of oligomeric esters is neglected).

For the 80% lactic acid solution used and the polymerization degree of oligomer $\bar{\mathrm{PD}}=10.25$, the water content of the system is reduced by *RW* = 95.67%. Compared to direct esterification carried out according to Figure 4, it is clear that water in the system, as one of the esterification products, directly affects the equilibrium composition of the mixture and, therefore, negatively affects the yield of ethyl lactate. Oligomeric ethyl esters and lactic acid oligomers are also produced in the real system. Oligomers together with water complicate the separation of pure ethyl lactate. Ethyl lactate forms an inseparable azeotrope with water resulting in product loss. In the case of direct esterification of 80% lactic acid with ethanol, this loss is theoretically equal to 14.7% of the product.

The main purpose of LA condensation was to limit the amount of water in the system. The mass fraction of residual “free” water in the oligomer product was also monitored with respect to the degree of polymerization. This free water directly affects the rate and achievable degree of condensation. At the higher polycondensation degree, the residual free water can no longer be removed by distillation due to a quick increase in viscosity. Complete removal of free water represents a technical barrier for the realization of direct polycondensation of lactic acid to the high molecular weight polymer. The free water content was monitored by titration of the taken samples by the Karl Fischer method.

**Figure 5:**
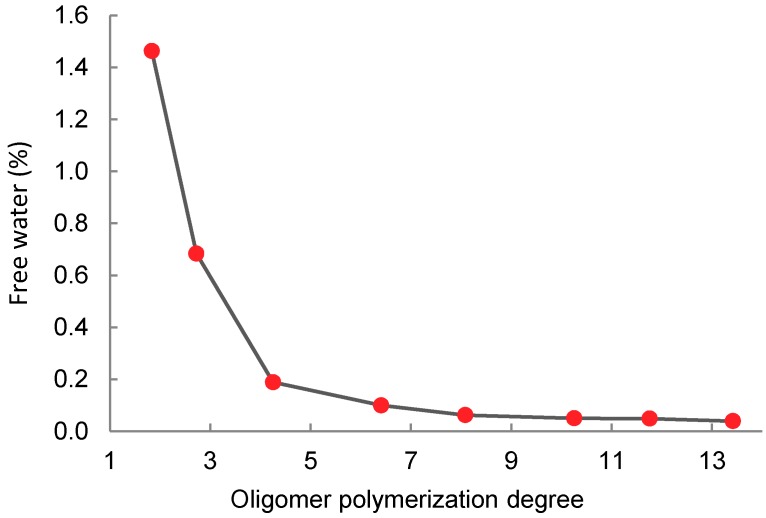
Free water fraction in the oligomeric lactic acid depending on the polymerization degree.

Figure 5 shows that even if reaching the average polymerization degree of 4 or more, the content of free water in the oligomer (relative to the original 20% in the lactic acid solution) is significantly reduced. The subsequent sharp drop at $\bar{\mathrm{PD}}=4$ is due to the progressive reduction of pressure in the polycondensation reactor (Figure 3). At $\bar{\mathrm{PD}}=10.25$, the free water content is 0.049%. The bound water releasable by oligomer end-group esterification (Figure 1 and Equation (16)) represents 4.33% as compared to direct esterification of 80% LA. The water content of the esterification system is reduced more than 20 times by polycondensation. The oligomer thus prepared was used for further experiments with transesterification of the oligomer with ethanol to ethyl lactate. Some properties of the prepared oligomer are shown in the Table 1.

### 2.4. Transesterification of Oligomer with Ethanol

**Figure 6:**
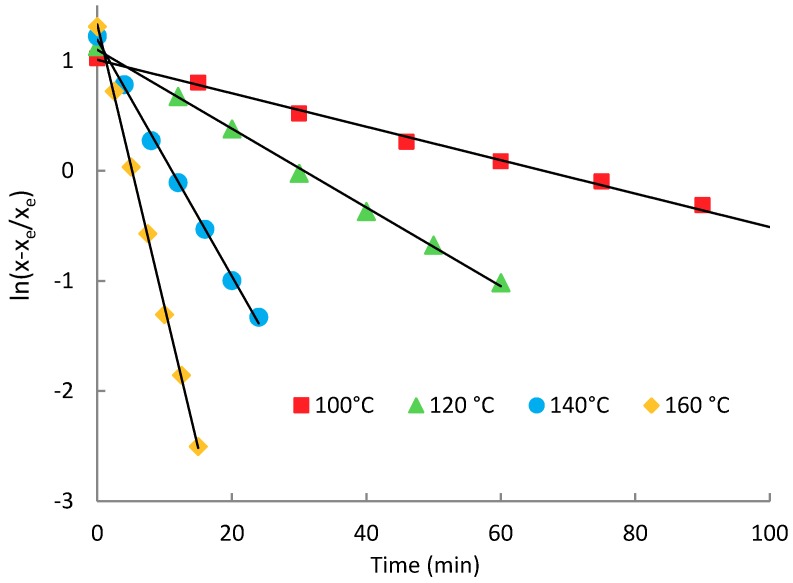
Linearized dependence of ethanol concentration on time during the transesterification of oligomeric lactic acid with ethanol.

**Figure 7:**
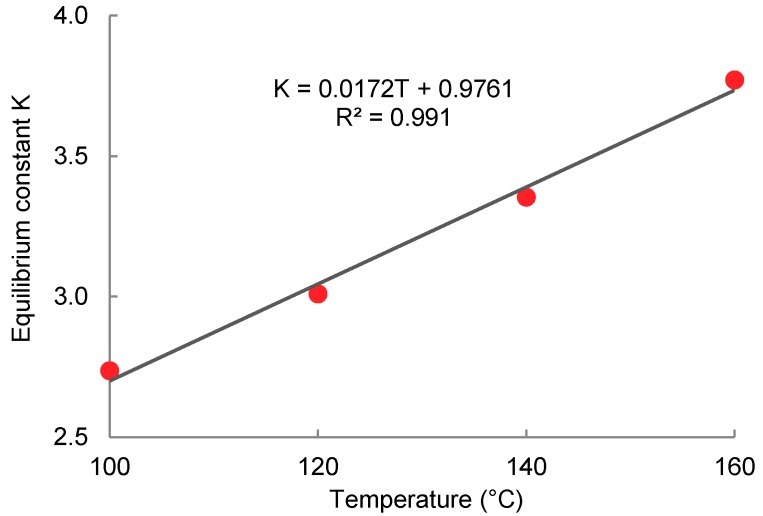
Dependence of the equilibrium constant for oligomeric lactic acid transesterification on the ethanol temperature.

The data obtained of ethanol concentration change over the time for different alcoholysis temperatures were plotted in the linearized form according to Equation (14) and are shown in Figure 6. The iterative calculation was used to obtain the model parameters. The free ethanol concentration at equilibrium (Figure 1) *x_e_* was set so that the deviation from the linear trend was minimal. The Pearson correlation coefficient (r^2^) was used as the iteration parameter and *x_e_* was adjusted to get r^2^ as close to 1 as possible. When the equilibrium concentration was adjusted, the equilibrium constant *K* was calculated from the intercept of the linearized trend according to Equation (14) and the overall rate constant $\overset{↔}{\;k}$. Linearized data according to the model equation show a very close correlation with the linear trend, as indicated by the correlation coefficient values given in Table 2. The comparative experiment carried out under the same conditions with an equimolar amount of lactic acid in the form of 80% aqueous solution instead of oligomer shows a much faster rate of esterification compared to the transesterification. The reaction rate was roughly 11 times greater under the same reaction conditions (100 °C). It is also apparent that the equilibrium composition of the mixture is affected by the presence of water, as shown by the equilibrium constant *K* and the conversion. The transesterification is an endothermic equilibrium process, as evidenced by the dependence of the equilibrium constant on the temperature. Its value increases with temperature roughly linearly, as shown in Figure 7. Dependence of the equilibrium composition (constants) on the temperature is based on the different temperature coefficients of the forward and reverse rate constants.

The measurements carried out at 180 °C were not further processed because of the presence of a significant experimental error due to short sampling intervals (1 min). Heating and cooling of the pressure reactor represented a substantial part of the reaction time.

**Figure 8:**
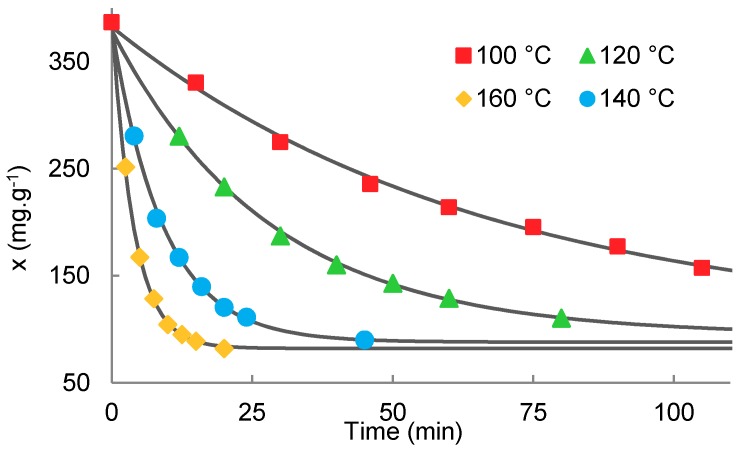
Time dependence of the ethanol concentration during the transesterification of oligomeric lactic acid with ethanol (marks—experimental data; curves—model according to Equation (15)).

Figure 8 shows the tight match of the model dependencies resulting from the finding of derived constants from Table 2 to Equation (17). It should be noted that the combination of purely kinetic variables, such as the rate constant of the reaction, with the equilibrium constant of thermodynamic nature needs to be used with great caution. This special case can be used only when both directions of the reaction show the same reaction mechanism, order of reaction, and the same stoichiometry. The esterification and transesterification of carboxylic acids with alcohol fulfill these conditions. The order of the reaction may change during the reaction course; therefore, reliable data can be obtained by measuring the reaction rate from the beginning to high conversion. The speed constants obtained using the initial velocity method do not provide reliable results.

Good consistency of the data obtained allowed the calculation of the activation energy of transesterification. The activation energy and the pre-exponential factor were obtained from the Arrhenius equation in a linearized form:

(17) $$\ln\left(k\right)=\frac{-E_{a}}{R}\cdot \frac{1}{T}+\ln\left(A\right)$$

**Figure 9:**
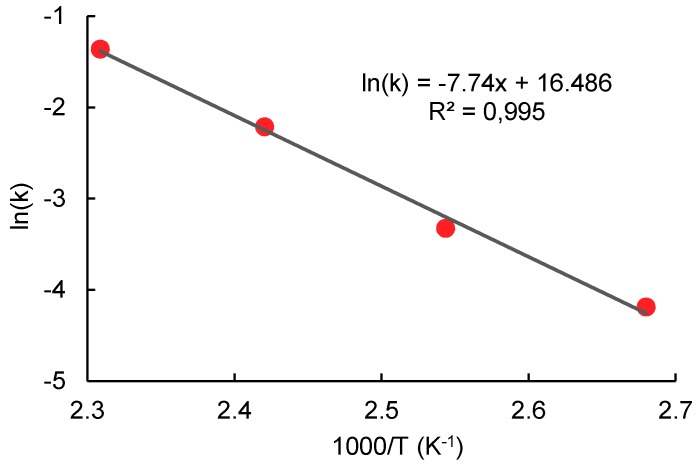
Arrhenius plot of the rate constant dependency on the reciprocal value of thermodynamic temperature.

The values of the activation energy and pre-exponential factor of the transesterification obtained by the linear regression of data shown in Figure 9 are equal to E_a_ = 64.35 kJ·mol^−1^; A = 1.445∙10^7^·s^−1^.

## 3. Experimental

### 3.1. Materials

Food grade lactic acid, 80% water solution, L-isomer > 98%, Fichema, Brno, Czech Rep. Anhydrous ethanol p.a., Lachema, Brno, Czech Rep.; Anhydrous iron(III) chloride p.a., Lach-Ner, Neratovice, Czech Rep.; Anhydrous methanol, Lach-Ner, Neratovice, Czech Rep.; 1,4-dioxane p.a., Lach-Ner, Neratovice, Czech Rep.; Acetone p.a., Penta-chemicals, Chrudim, Czech Rep.; Bromthymol blue, Lachema, Brno, Czech Rep.; Hydrogen purity 99.997%, Siad, Praha, Czech Rep.; Potassium hydrogen phthalate p.a., Lach-Ner, Neratovice, Czech Rep.; Two-component Karl Fischer reagents based on pyridine and SO_2_ in methanol, Penta-chemicals, Chrudim, Czech Rep.

### 3.2. Oligomeric LA Preparation

Total of 1.8 kg of 80% aqueous solution of lactic acid was auto-catalytically polycondensed in a 2.5 dm^3^ double-jacketed glass reactor. The jacket temperature was kept constant at 165 °C throughout the condensation. The reactor was equipped with an anchor stirrer and stirred at a rate of 600 rpm. The reactor lid was equipped with a thermometer, sampling port, and a 20 cm long, glass packed column. A spiral cooler was connected to the column. The condensate was collected in a round two-neck flask attached to a vacuum gauge and vacuum pump. In the first two hours the condensation was carried out at atmospheric pressure and then the pressure in the reactor was gradually reduced to 2 kPa. The maximum temperature was limited by the jacket and was not regulated during the process. The reaction samples were taken in hourly intervals. The samples were titrated to determine the number average molecular weight and free water content. The pressure, temperature, and number average molecular weight progress during the condensation are documented in Figure 3. Upon the completion of condensation, the oligomer was drained into a silicone bowl at about 100 °C and cooled to laboratory temperature. The glassy oligomer was disintegrated to coarse particles and stored in a desiccator.

### 3.3. Oligomer Alcoholysis

The prepared lactic acid oligomer was subjected to alcoholysis with ethanol under autogenous pressure. The reaction mixture for the experimental alcoholysis was prepared in one run for all samples representing one series (reaction temperature). Then, 20 g of oligomeric lactic acid (0.270 mol of lactate units) was melted at 120 °C in a 50 mL round-bottom flask equipped with a reflux condenser and magnetic stirrer. Anhydrous ethanol was added to the melt through the condenser in an equimolar amount with respect to lactate units of oligomer (i.e., 0.207 mol, 12.54 g). Anhydrous ferric chloride in the amount of 1 mol% (0.43 g) was dissolved in ethanol as an alcoholysis catalyst before the addition. The oligomer was homogenized for 30 s with ethanol and the catalyst using a magnetic stirrer. The resulting homogeneous solution was rapidly cooled with ice water. The reaction mixture was transferred into glass test tubes (2.5 g) immediately after the preparation. The test tubes were tightly sealed in brass pressure containers, allowing the work at elevated pressure. A total of eight samples was simultaneously immersed in a glycerol circulating thermostat bath. At given time intervals, the individual pressure containers were removed and cooled in ice water. The samples were diluted with 1,4-dioxane in a weight ratio of 1:1 after the alcoholysis. Dioxane acts both as a solvent for the crystalline part of the oligomer and as an internal standard to determine the concentration of free ethanol and ethyl lactate by GC. The measurement was performed for reaction temperatures of 100–180 °C with 20 °C steps and sampling intervals of 15; 7.5; 5; 2.5, and 1 min.

## 4. Instruments and Analysis

### 4.1. Determination of Molecular Weight and Polymerization Degree of Oligomer

The number average molecular weight $\bar{M_{n}}$ of lactic acid oligomers was determined by the acid-base titration of terminal carboxyl groups. The titration was performed in an acetone solution with bromothymol blue as an indicator. The sample amount was about 0.5 g. The standard solution was 0.1 mol·dm^−3^ KOH in methanol standardized with potassium hydrogen phthalate. The number average molecular weight corresponds to the relation:

(18) $$\bar{M_{n}}=\frac{m_{s}}{V_{t}\cdot c_{s}}\;\left[g\cdot {\mathrm{mol}}^{-1}\right]$$

where $m_{s}$ is the mass of the sample in grams, $V_{t}$ is the titration solution consumption in liters, and $c_{s}$ is the concentration of titration solution in mol·dm^−3^.

The number average degree of polymerization $\bar{PD}$ of oligomeric lactic acid corresponds to the relation:

(19) $$\bar{PD}=\frac{\bar{M_{n}}-M_{H_{2}O}}{M_{\mathrm{LAU}}}\;$$

where $M_{\mathrm{LAU}}=$ 72.08 g·mol^−1^ is the molecular weight of lactic acid repeating unit, $M_{H_{2}O}=$ 18 g·mol^−1^ is the molecular weight of water (correction of end groups).

### 4.2. Ethanol and Ethyl Lactate Concentration Determination by Gas Chromatography

The ethanol and ethyl lactate concentrations were determined by gas chromatography using fast GC system of our construction (90 s per sample analysis). The manual sample injection of 0.2 μL with 1,4-dioxane as an internal standard (1:1 by mass mixture with sample) was performed. The column was 2.2 m long with an inner diameter of 2.1 mm. The stationary phase was 10% Carbowax 20 M on a Chromosorb W-AW, 80–100 mesh. The carrier gas was hydrogen at a flow rate of 15 cm^3^·min^−1^. TCD detection was used and the isothermal column thermostat was set at 135 °C. The calibration was with ethanol and ethyl lactate standards with the addition of the internal standard 1,4-dioxane. The concentrations of ethanol and ethyl lactate in the reaction mixture were determined simultaneously on the basis of linear calibration dependencies (six standards for each of the components). For each sample series corresponding to one reaction temperature, it was necessary to replace the injector liner due to accumulation of non-volatile oligomeric residue and the catalyst contained in the samples. Samples were injected without further treatment or derivatization.

### 4.3. Melting Point of the Oligomer

The melting point of the oligomer was determined using DSC (TA Instruments 2920, New Castle, DE, USA). The sample (10 mg) was measured in an aluminum pan. The first scan was used to determine the melting temperature. Before the measurement, the sample was crystallized for 30 min at 80 °C. The heating rate was 10 °C/min^−1^ in the range of 30–190 °C.

### 4.4. Determination of Water Content

The water content of the oligomer samples and ethyl lactate was determined by the Karl Fischer method. The volumetric titration unit of our construction with the biamperometric endpoint detection was used. The titrating solution was standardized with sodium tartrate dihydrate. The titration was carried out in a 1:1 mixture of methanol and chloroform. Used sample weight was in the range of 0.1–1 g with respect to the water content.

## 5. Conclusions

The kinetic model for the transesterification of polyester with alcohol with respect to alcohol sufficiently reflects the real behavior of the system, even in the case of transesterification of the oligomer of a relatively small chain length. The verification of the model with the example of oligomeric lactic acid alcoholysis with ethanol demonstrated the advantage of this previously unused route of preparation of anhydrous ethyl lactate. By the pre-oligomerization of lactic acid, the reaction medium is substantially free of water. The subsequent alcoholysis to ester takes place already in an anhydrous environment. The anhydrous environment allows the use of a variety of catalysts that otherwise hydrolyze. By removing water, the chemical equilibrium is also greatly shifted to the formation of ethyl lactate. Achieving a good yield of ethyl ester is thus already possible with molar equivalence of reactants. Anhydrous ferric chloride was experimentally confirmed as an appropriate and environmentally friendly transesterification catalyst. This strong Lewis acid cannot be used for direct esterification of lactic acid because of rapid hydrolysis in water. The pre-oligomerization step, therefore, expands the possible selection of catalysts. The pre-oligomerization step, in fact, does not affect the energy balance of the ethyl lactate preparation since it does not matter whether the water is removed from the mixture before or after ester formation. The energy required to evaporate the water during the distillation step is constant.

## Figures and Tables

**Table 1:** Oligomeric lactic acid properties.

Property of Oligomer	Value
$\bar{M_{n}}$	758.4 g·mol^−1^
$\bar{\mathrm{PD}}$	10.25
Melting point	103.4 °C
Yield	99.8%
Free water	0.049%
Lactic acid in condensate	0.17

**Table 2:** Kinetic parameters of oligomeric lactic acid alcoholysis with respect to ethanol.

Temperature (°C)	x_0_ (mg·g^−1^)	$\overset{↔}{k}\;(\min-1)$	K	x_e_ (mg·g^−1^)	r^2^	Half-Time (min)	Conversion (%)
100 (LA) *	290	−0.1643	0.921	148.00	0.995	4.22	60.8
100	387	−0.0152	2.736	102.44	0.996	45.66	72.9
120	387	−0.0359	3.009	94.67	0.999	19.28	75.0
140	387	−0.1093	3.354	88.03	0.999	6.34	76.7
160	387	−0.2563	3.771	82.19	0.999	2.70	78.3
180	387	−0.3520	4.827	66.99	0.997	1.97	82.3

* comparative values for the experiment with 80% lactic acid instead of oligomer.
